# Cardiometabolic comorbidities and prostate cancer risk: evidence from the prostate, lung, colorectal, and ovarian cancer screening trial

**DOI:** 10.3389/fonc.2026.1799790

**Published:** 2026-04-29

**Authors:** Qian Zhu, Lan Zeng, Menghui Chen

**Affiliations:** 1 Key Laboratory of Cardiomyopathy and Heart Failure, Affiliated Hospital of Panzhihua University, Sichuan, China; 2 Clinical Medical Research Center, Panzhihua Central Hospital, Sichuan, China; 3 Department of Geriatric Medicine, Panzhihua Central Hospital, Sichuan, China

**Keywords:** cardiometabolic diseases, comorbidity, diabetes, prostate cancer, specific mortality

## Abstract

**Background:**

This study aims to explore the association between cardiometabolic diseases (CMDs) involving diabetes and the risk of prostate cancer (PCa) as well as its mortality.

**Methods:**

The population-based cohort, comprising 71, 566 participants, was derived from the Prostate, Lung, Colorectal, and Ovarian (PLCO) Cancer Screening Trial. CMDs encompassed three conditions, namely diabetes, heart diseases, and stroke. Multivariable Cox proportional hazards and competing risk regression models were used to estimate hazard ratios (HRs) and 95% confidence intervals (CIs) for assessing associations between individual diabetes, coexisting CMDs, and PCa incidence and mortality.

**Results:**

At baseline, 12, 979 participants had at least one CMD, while 2, 391 had two or more concurrent CMDs. Over a 17-year mean follow-up, 8, 263 participants were diagnosed with PCa. Participants with baseline diabetes exhibited an 18% elevated risk of developing PCa compared with CMD-free counterparts (HR: 1.18, 95% CI: 1.08~1.29). Among participants with multimorbidity, only diabetes-heart disease comorbidity was significantly associated with an elevated risk of PCa incidence (HR: 1.31, 95% CI: 1.09~1.59). Neither diabetes, heart disease, nor stroke had a statistically significant association with PCa-specific mortality risk. However, compared with CMD-free individuals, those with a single type of CMD exhibited a significantly reduced risk of non-aggressive PCa-specific mortality (HR: 0.50, 95% CI: 0.26~0.99).

**Conclusions:**

Baseline diabetes was independently associated with an increased incidence of PCa, and the comorbidity of diabetes with heart disease further amplified this risk. The presence of a single CMD reduced the risk of non-invasive PCa-specific mortality in affected individuals.

## 1 Introduction

Currently, the disease burden of population is shifting from premature death to long-term illness, and “multimorbidity” is emerging as the next epidemic in non-communicable diseases. Prostate cancer (PCa) was the second most common malignant tumor among men worldwide (1). Clinically, approximately 40% of PCa patients have at least one comorbidity (2, 3), among which cardiovascular diseases, obesity and metabolic diseases, and urinary diseases are the most common (4, 5). Possible interactions between PCa and different comorbid clinical entities can influence clinical care of patients, with interventions having positive, antagonistic, or neutral effects due to heterogeneity of disease associations (6, 7). Therefore, evaluation of possible interactions between PCa and different comorbidity entities is important for patient management.

Cardiometabolic diseases (CMDs) refer to a large category of diseases accompanied by metabolic disorders and cardiovascular diseases, including diabetes, coronary heart disease and stroke, etc. Patients with cardiovascular diseases have a significantly increased risk of PCa, especially atherosclerotic cardiovascular diseases (8). Published meta-analyses have shown inconsistent and conflicting results regarding the association between diabetes and PCa risk, which may be influenced by geographical region, medication use, and age at diabetes onset (9–11). Specifically, diabetes was associated with a lower risk of PCa among European populations but an increased risk among Asian populations (9, 12). Furthermore, insulin therapy and early-onset diabetes were associated with a reduced risk of PCa (11). At present, there is not enough evidence to support the causal assertion that stroke is associated with PCa. Only one Mendelian randomization study indicated that stroke may exert a protective effect against PCa (13). Chronic diseases may be regarded as “precondition” for the occurrence of cancer. However, existing studies have mostly focused on the association mechanisms between individual CMDs and PCa. What still needs to be further elucidated is whether the synergistic effects of multiple concurrent CMDs exert a cumulative impact on PCa, whether there is heterogeneity in the associations between different types of CMDs and PCa, and how such heterogeneity modulates PCa risk in the context of multimorbidity.

Therefore, in this study, based on the longitudinal follow-up cohort of the Prostate, Lung, Colorectal, and Ovarian (PLCO) Cancer Screening Trial, and in accordance with the baseline CMDs registered by the participants, we used the Cox proportional hazards regression and competing risk regression models to evaluate the association between CMDs and PCa incidence and PCa-specific mortality risk, and further evaluated the independent impact of each disease or CMD combination on the risk of PCa.

## 2 Materials and methods

### 2.1 Study design and participants

**Figure 1:**
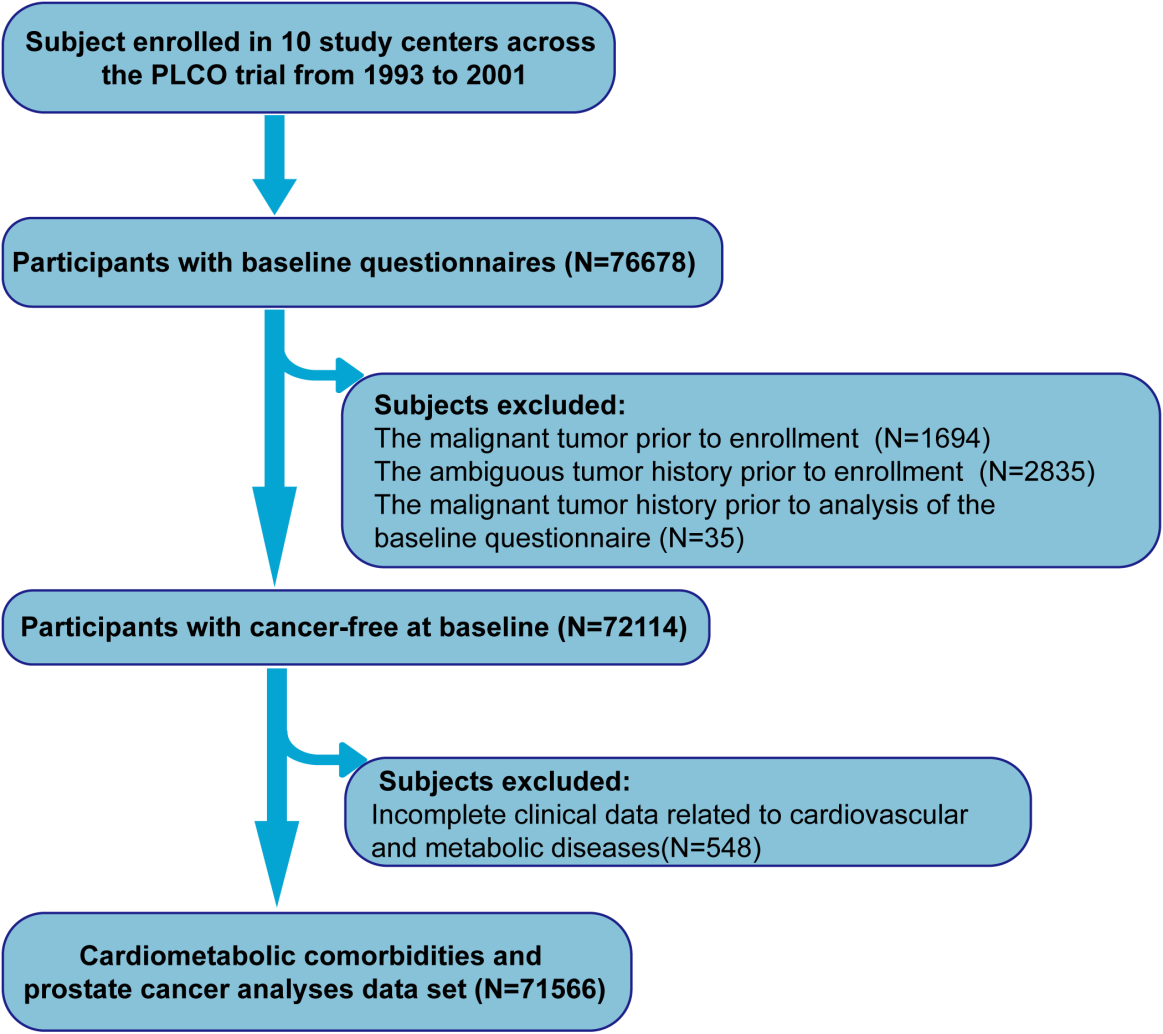
The flow chart of identifying individuals eligible for our study.

The data for this study were obtained from the PLCO Cancer Screening Trial and study design of trial has been reported elsewhere (14). Specifically, eligible participants aged between 55 and 74 years were recruited from November 1993 through September 2001 and randomly assigned to either the screening group or the control group for investigating whether screening could decrease the risk of mortality from cancers. The study subjects adhered to the following exclusion criteria: having an unretrieved baseline questionnaire or invalid data; having been diagnosed with a malignant tumor or having an ambiguous tumor history prior to enrollment; having a history of cancer before the analysis of the baseline questionnaire; having incomplete clinical data related to cardiovascular and metabolic diseases. The detailed process is shown in **Figure 1**. As this study was based on a secondary analysis of the existing PLCO cohort, *a priori* sample size and power calculation was not performed. The large sample size (N = 71, 566) and sufficient number of outcome events were considered to have provided sufficient statistical power in this study to detect the observed associations.

### 2.2 Ethical considerations

The ClinicalTrials.gov registration numbers of prostate cancer screening trial with PLCO: NCT00002540. The data for this study were obtained from the PLCO trial. The original study received approval from the relevant ethics committee, and informed consent was obtained from all participants prior to the research. Our study utilized anonymized data from this database and no separate ethical approval is required.

### 2.3 Exposure variable

CMDs encompassed three conditions, namely diabetes, heart diseases, and stroke. Heart diseases include hypertension, myocardial infarction, atrial fibrillation, ischemic heart disease, coronary artery disease, and congestive heart failure. Stroke includes ischemic stroke, hemorrhagic stroke, and transient ischemic attack. This composite definition may mask differential associations across individual cardiovascular conditions. The diagnosis of these disorders relies on the self-reported medical history of individuals. Participants were classified according to the total number of CMDs present: none, single, or multiple cardiometabolic multimorbidity (CMM). CMM was defined as the coexistence of two or more concurrent CMDs. Furthermore, CMM was subdivided into the following eight combinations: CMD-free; only diabetes; only heart diseases; only stroke; diabetes and heart diseases; heart diseases heart diseases and stroke; diabetes and stroke; coexistence of all three conditions. Participants were classified as CMD-free if they had none of diabetes, heart disease, or stroke. Single CMD was defined as the presence of one CMD only. Only diabetes: diabetes without heart disease or stroke. Only heart disease: heart disease without diabetes or stroke. Only stroke: stroke without diabetes or heart disease.

### 2.4 Outcomes

The study outcomes comprised PCa incidence and PCa- specific mortality risk. Throughout the follow-up period, newly diagnosed PCa cases were ascertained and verified via annually updated questionnaires. Time-to-event was calculated as the interval between the date of study enrollment and the date of PCa diagnosis. For participants with suspected or self-reported PCa, medical records were retrieved for further diagnostic confirmation, with details on tumor stage, grade and pathological characteristics also extracted. PCa cases were further stratified into two subgroups based on clinical and pathological features: Non-aggressive PCa: defined as tumors with a Gleason score ≤ 6 and clinical stage T1~T2a; Aggressive PCa: defined as tumors with a Gleason score ≥ 7, and/or clinical stage ≥ T2b, or with distant metastasis. Death events were identified and verified using reports from immediate relatives, official death certificates and autopsy records (where available). PCa-specific survival was defined as the time interval from the date of PCa diagnosis to the date of death attributable to PCa.

### 2.5 Statistical analysis

To minimize the potential biases and maximize the statistical power, we used the following approaches to impute missing values of 13 variables. Given the low proportion of missing data (<5%), single imputation using the median for continuous variables and the mode for categorical variables was considered appropriate. **Supplementary Table 1** presents the distribution of variables with missing values before and after data imputation.

The baseline characteristics of participants were described as number (percentage) or mean ± SD. Chi-square and one-way ANOVA tests were used to compare the between-group differences. Kaplan-Meier survival analysis was utilized to quantify the median survival duration and cumulative survival probabilities for each independent variable.

Cox regression models with time-dependent covariates (*coxph* function from *survival* package) were used to estimate the HRs and 95% CIs of association between baseline CMDs and risk of PCa development during the follow-up period. Competing risk regression analysis (*crrFormula* function from the *cautoReg* package) was employed to estimate the HR (95% CI) between baseline CMDs and PCa- specific mortality risk. Unadjusted model served as the baseline model without any variable adjustments. Adjusted model incorporated demographic characteristics (age, education, occupation, and marital status), and potential confounding factors such as body mass index (BMI), behavioral risk factors, first-degree relatives with cancer, prior PSA testing and physical condition.

Statistical analyses were conducted by using SPSS and R version 4.4.3. All tests in this study were two-sided with a significance level of *P valu*e < 0.05.

## 3 Results

### 3.1 Characteristics of the study subjects

Among 71, 566 participants who met the inclusion criteria, 12, 979 participants had at least one CMD at baseline, while 2, 391 had two or more concurrent CMDs. Those with CMM tended to be older on average and have a greater percentage of retirees and low-education. Furthermore, Patients with CMM were more likely to be former smokers, possess higher BMI and prevalence of multiple diseases, including hypertension, lung diseases, liver comorbidity, arthritis and osteoporosis (**Table 1**).

**Table 1:** Baseline characteristics of study population according to baseline CMD status^a^.

Characteristics	CMD status			
	None	Single CMD	CMM	*P-value*
	56,196	12,979	2,391	
Baseline age				
≤59	19,634(34.9)	3,006(23.2)	420(17.6)	<0.001
60-64	17,926(31.9)	3,885(29.9)	672(28.1)	
65-69	12,221(21.7)	3,622(27.9)	748(31.3)	
≥70	6,415(11.4)	2,466(19.0)	551(23.0)	
(Mean, SD)	62.26 ± 5.23	64.07 ± 5.36	64.99 ± 5.20	<0.001
Race				
white	49,965(88.9)	11,129(85.7)	2,034(85.1)	<0.001
non-white	6,209(11.0)	1,830(14.1)	356(14.9)	
preferred not to answer	22(0.0)	20(0.2)	1(0.0)	
Education level				
college below	21,040(37.4)	5,637(43.4)	1,107(46.3)	<0.001
college/postgraduate	35,156(62.6)	7,342(56.6)	1,284(53.7)	
Occupation				
working	26,872(47.8)	4,267(32.9)	505(21.1)	<0.001
retired	26,430(47.0)	7,697(59.3)	1,575(65.9)	
others b	2,894(5.2)	1,015(7.8)	311(13.0)	
Marital status				
married	46,571(82.9)	10,738(82.7)	1,941(81.2)	<0.001
widowed	1,896(3.4)	554(4.3)	135(5.6)	
divorced	5,178(9.2)	1,111(8.6)	194(8.1)	
separated	611(1.1)	148(1.1)	42(1.8)	
never married	1,940(3.5)	428(3.3)	79(3.3)	
Body mass index, BMI (kg/m^2^)				
<18.5	247(0.3)	192(0.3)	7(0.3)	<0.001
18.5 to <25.0	18,706(26.1)	15,499(27.6)	428(17.9)	
25.0 to <30.0	36,594(51.1)	29,027(51.7)	1,129(47.2)	
≥30.0	16,019(22.4)	11,478(20.4)	827(34.6)	
(Mean, SD)	27.26 ± 3.97	28.23 ± 4.50	28.94 ± 4.80	<0.001
Smoking status				
current	21,628(38.5)	3,860(29.7)	675(28.2)	<0.001
former	6,692(11.96)	1,473(11.3)	223(9.3)	
never	27,876(49.6)	7,646(58.9)	1,493(62.4)	
Pack-years of cigarette smoking				
never (0)	21,628(38.5)	3,860(29.7)	675(28.2)	<0.001
>0 and ≤20	11,558(20.6)	2,185(16.8)	337(14.1)	
>20	23,010(40.9)	6,934(53.4)	1,379(57.7)	
First-degree relatives with cancer				
never	27,003(48.1)	6,293(48.5)	1,191(49.8)	0.182
yes	29,193(51.9)	6,686(51.5)	1,200(50.2)	
Aspirin				
no	29,791(53.0)	3,866(29.8)	507(21.2)	<0.001
yes	26,405(47.0)	9,113(70.2)	1,884(78.8)	
Ibuprofen				
no	42,868(76.3)	10,188(78.5)	1,914(80.1)	<0.001
yes	13,328(23.7)	2,791(21.5)	477(19.9)	
Enlarged prostate or BPH				
no	44,542(79.3)	9,836(75.8)	1,752(73.3)	<0.001
yes	11,654(20.7)	3,143(24.2)	639(26.7)	
Prior PSA test				
no	26,157(46.5)	5,529(42.6)	1,066(44.6)	<0.001
yes-one	20,133(35.8)	4,658(35.9)	795(33.2)	
yes-more than one	5,356(9.5)	1,408(10.8)	225(9.4)	
does not know	4,550(8.1)	1,384(10.7)	305(12.8)	
Hypertension				
no	39907(71.0)	6383(49.2)	779(32.6)	<0.001
yes	16289(29.0)	6596(50.8)	1612(67.4)	
Lung diseases c				
no	53,259(94.8)	11,982(92.3)	2,156(90.2)	<0.001
yes	2,937(5.2)	997(7.7)	235(9.8)	
Colon_commodity				
no	55,591(98.9)	12,796(98.6)	2,345(98.1)	<0.001
yes	605(1.1)	183(1.4)	46(1.9)	
Liver_commodity				
no	53,997(96.1)	12,393(95.5)	2,269(94.9)	<0.001
yes	2,199(3.9)	586(4.5)	122(5.1)	
Arthritis				
no	40,532(72.12)	8,322(64.1)	1,346(56.3)	<0.001
yes	15,664(27.9)	4,657(35.9)	1,045(43.7)	
Osteoporosis				
no	55,796(99.3)	12,838(98.9)	2,352(98.4)	<0.001
yes	400(0.7)	141(1.1)	39(1.6)	

^a^ N= 71566. Values are mean ± SD for continuous variables and counts (percentage) for categorical variable as indicated.

^b^ “Others” refers to homeworker, unemployed, extended sick leave and disabled.

^c^ “Lung diseases” refers to asthma, chronic obstructive pulmonary disease (COPD), and bronchiectasis.

BMI, body mass index; CMD, cardiometabolic disease; CMM, cardiometabolic comorbidities; BPH, benign prostatic hyperplasia.

**Figure 2:**
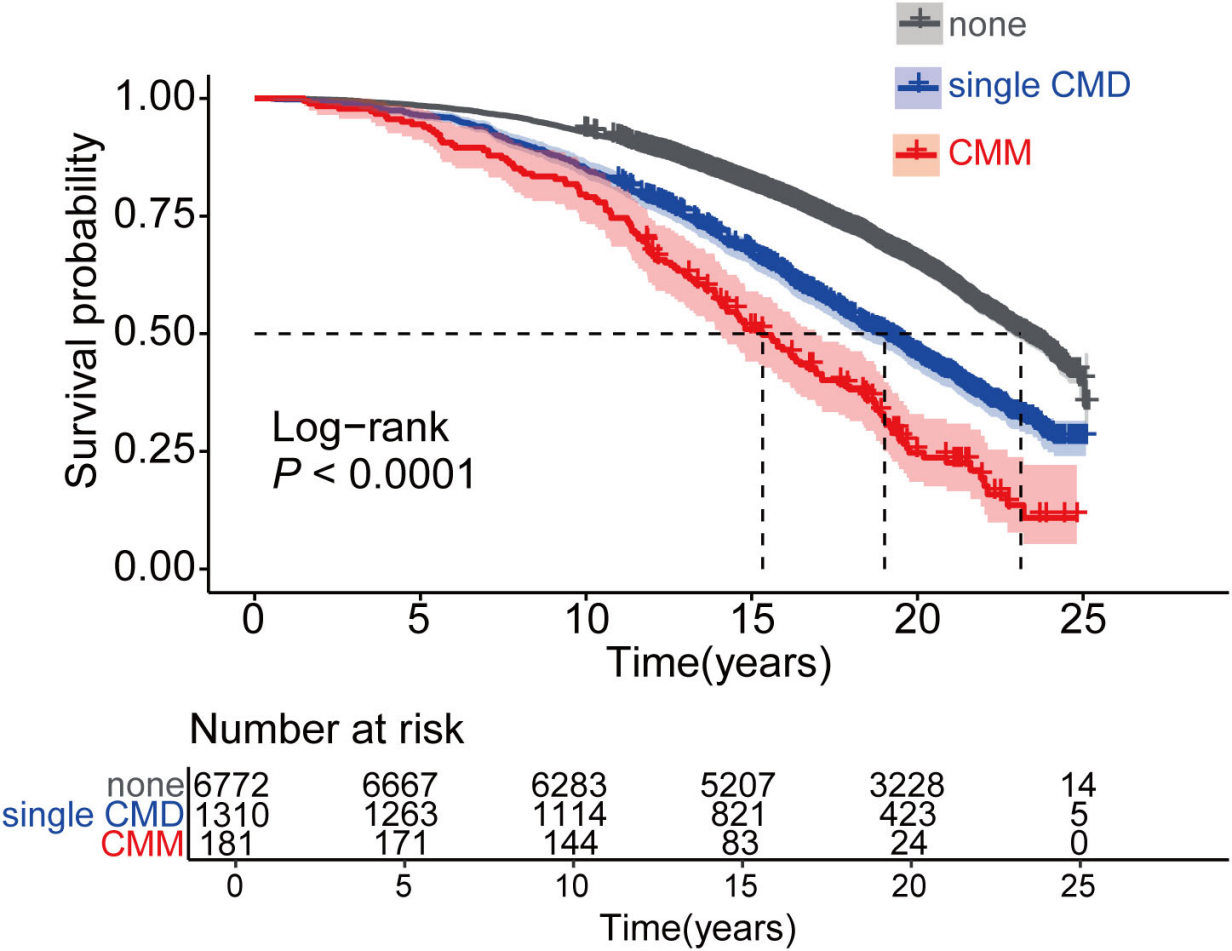
Kaplan–Meier curves of cumulative incidence of PCa survivor’s death according to categories of CMD status.

During a median follow up of 17 years, 8263 cases of PCa occurred, including 6772 cases in CMDs-free individuals, 1310 cases in the single CMD individuals and 181 cases in CMM individuals, respectively (**Supplementary Table 2**). Notably, individuals with baseline CMM presented a greater proportion of high AJCC stage, cancer grade, and Gleason scores when developing PCa, as well as higher mortality rate and shorter median survival time (**Supplementary Table 3**, **Figure 2**).

### 3.2 Associations between baseline CMDs and risk of PCa incidence

In the unadjusted model, participants with baseline diabetes had a 21% higher risk of developing PCa compared with CMD-free counterparts (HR: 1.21, 95% CI: 1.11~1.32). This positive association between diabetes and PCa risk remained statistically significant after full adjustment for potential confounding factors (HR: 1.18, 95% CI: 1.08~1.29). Participants with stroke without diabetes or heart diseases had a 27% increased risk of PCa incidence (HR: 1.27, 95% CI: 1.06~1.54). In contrast, although single CMD (HR: 1.07, 95% CI: 1.01~1.14) and CMM (HR: 1.20, 95% CI: 1.04~1.40) were linked to elevated PCa incidence risk in the unadjusted model, their direct effects attenuated and no longer reached statistical significance following full confounder adjustment. Among participants with two or more concurrent CMDs, only the diabetes-heart disease comorbidity was significantly associated with an increased risk of PCa incidence (HR: 1.31, 95% CI: 1.09~1.59) (**Table 2**).

**Table 2:** Association of baseline CMD with PCa incidence.

Exposure	N	Cases	Unadjusted model		Adjusted model	
			HR (95%CI)	*P-value*	HR (95%CI)	*P-value*
Diabetes						
no	65,091	7,733	1.00 (reference)		1.00 (reference)	
yes	6,475	530	1.21(1.11, 1.32)	<0.001	1.18(1.08, 1.29)	<0.001
Heart diseases						
no	62,033	7,291	1.00 (reference)		1.00 (reference)	
yes	9,533	972	1.20(1.04, 1.39)	0.014	0.97(0.91, 1.04)	0.393
Stroke						
no	69,629	8,082	1.00 (reference)		1.00 (reference)	
yes	1937	181	1.02(0.95, 1.09)	0.628	1.11(0.96, 1.29)	0.164
CMM status						
none	56,196	6,772	1.0(reference)		1.0(reference)	
single CMD	12,979	1,310	1.07(1.01, 1.14)	0.020	1.03(0.97, 1.09)	0.404
CMM	2,391	181	1.20(1.04, 1.40)	0.014	1.13(0.97, 1.31)	0.110
CMD combination						
none	56,196	6,772	1.0(reference)		1.0(reference)	
only diabetes	4,574	394	1.20(1.09, 1.33)	<0.001	1.17(1.05, 1.29)	0.003
only heart diseases	7,375	805	0.99(0.92, 1.06)	0.781	0.95(0.88, 1.03)	0.212
only stroke	1,030	111	1.38(1.14, 1.66)	<0.001	1.27(1.06, 1.54)	0.012
diabetes and heart diseases	1,484	111	1.35(1.12, 1.63)	0.002	1.31(1.09, 1.59)	0.005
diabetes and stroke	233	14	0.72(0.43, 1.22)	0.225	0.69(0.41, 1.17)	0.170
heart diseases and stroke	490	45	1.07(0.80, 1.44)	0.644	0.97(0.72, 1.30)	0.813
diabetes, heart diseases and stroke	184	11	1.57(0.97, 2.83)	0.135	1.49(0.82, 2.70)	0.188

Hazard ratios and 95% CIs were calculated using the Cox proportional hazards regression model. Adjusted model was adjusted for baseline age, education levels, occupation and marital status, body mass index, smoking status, first-degree relatives with cancer, prior PSA test, screening intervention and history of enlarged prostate or BPH, hypertension and lung diseases.

CMD, cardiometabolic disease; CMM, cardiometabolic comorbidities; HR, hazard ratio; CI: confidence interval; PCa, prostate cancer.

When stratified by PCa risk category, diabetes was significantly correlated with the occurrence of both non-aggressive (HR: 1.21, 95% CI: 1.04~1.40) and aggressive PCa (HR: 1.17, 95% CI: 1.04~1.31). There was a difference in the association between stroke and the risk of PCa with different aggressiveness, and a significant association was only observed in aggressive PCa (HR: 1.23, 95% CI: 1.01~1.51). After excluding the confounding effects of diabetes and heart disease, a significant positive correlation was found between the “stroke only” population and the risk of both types of PCa, with a stronger association strength. Notably, the diabetes-heart disease comorbidity still conferred a significantly elevated incidence risk for both aggressive and non-aggressive PCa subtypes (**Table 3**).

**Table 3:** Association of baseline CMD with incidence of PCa with different risks.

Exposure	Non-aggressive PCa			Aggressive PCa		
	Case	HR (95%CI)	*P-value*	Case	HR (95%CI)	*P-value*
Diabetes						
no	3070	1.00 (reference)		4663	1.00 (reference)	
yes	196	1.21(1.04, 1.40)	0.013	334	1.17(1.04, 1.31)	0.009
Heart diseases						
no	2852	1.00 (reference)		4439	1.00 (reference)	
yes	414	1.10(0.99, 1.23)	0.074	558	0.92(0.84, 1.01)	0.089
Stroke						
no	3182	1.00 (reference)		4900	1.00 (reference)	
yes	84	1.05(0.84, 1.31)	0.665	97	1.23(1.01, 1.51)	0.043
CMM status						
none	2657	1.00 (reference)		4115	1.00 (reference)	
single CMD	530	1.15(1.04, 1.26)	0.005	780	0.99(0.92, 1.08)	0.890
CMM	79	1.10(0.88, 1.38)	0.406	102	1.16(0.95, 1.43)	0.139
CMD combination						
none	2657	1.00 (reference)		4115	1.00 (reference)	
only diabetes	137	1.24(1.04, 1.48)	0.017	257	1.13(1.00, 1.29)	0.047
only heart diseases	343	1.09(0.97, 1.23)	0.136	462	0.91(0.82, 1.01)	0.050
only stroke	50	1.36(1.02, 1.82)	0.040	61	1.37(1.07, 1.77)	0.014
diabetes and heart diseases	45	1.46(1.07, 1.97)	0.015	66	1.28(1.00, 1.64)	0.048
diabetes and stroke	8	0.52(0.26, 1.04)	0.065	6	1.03(0.46, 2.29)	0.952
heart diseases and stroke	20	0.91(0.59, 1.42)	0.681	25	1.03(0.69, 1.53)	0.890
diabetes, heart diseases and stroke	6	1.93(0.86, 4.31)	0.110	5	1.17(0.48, 2.83)	0.724

### 3.3 Associations between baseline CMDs and PCa- specific mortality

We analyzed the association between individual CMDs, as well as their comorbid patterns, and PCa- specific mortality risk based on 8, 263 PCa cases. Results derived from the competing risk regression model showed that none of diabetes, heart disease, or stroke had a statistically significant association with PCa-specific mortality. Specifically, compared with CMD-free individuals, those with a single CMD exhibited a markedly reduced risk of non-aggressive PCa-specific mortality (HR: 0.50, 95% CI: 0.26~0.99). No statistically significant differences were observed in PCa-specific mortality risk across different PCa subgroups in relation to distinct CMD comorbid patterns (**Table 4**).

**Table 4:** Association of baseline CMD with PCa-specific mortality^a^.

Exposure	N	All PCa			Non-aggressive PCa			Aggressive PCa		
		Case	HR (95%CI)	P-value	Case	HR (95%CI)	P-value	Case	HR (95%CI)	P-value
Diabetes										
no	7733	563	1(reference)		102	1(reference)		461	1(reference)	
yes	530	36	0.71(0.50, 1.01)	0.060	4	0.53(0.19, 1.44)	0.210	32	0.74(0.51, 1.08)	0.120
Heart diseases										
no	7291	521	1(reference)		96	1(reference)		425	1(reference)	
yes	972	78	0.98(0.76, 1.28)	0.900	10	0.71(0.36, 1.42)	0.330	68	1.04(0.78, 1.38)	0.800
Stroke										
no	8082	583	1(reference)		104	1(reference)		479	1(reference)	
yes	181	16	1.10(0.66, 1.83)	0.710	2	0.66(0.16, 2.70)	0.560	14	1.22(0.70, 2.12)	0.480
CMM status										
none	6772	481	1(reference)		93	1(reference)		388	1(reference)	
single CMD	1310	107	0.99(0.79, 1.24)	0.950	10	0.50(0.26, 0.99)	0.046	97	1.09(0.86, 1.39)	0.470
CMM	181	11	0.60(0.32, 1.13)	0.110	3	0.94(0.30, 2.96)	0.910	8	0.53(0.25, 1.12)	0.100
CMD combination										
none	6772	481	1(reference)		93	1(reference)		388	1(reference)	
only diabetes	394	28	0.80(0.54, 1.18)	0.250	2	0.36(0.09, 1.50)	0.160	26	0.87(0.58, 1.31)	0.520
only heart diseases	805	68	1.07(0.81, 1.41)	0.620	7	0.58(0.26, 1.30)	0.190	61	1.18(0.87, 1.59)	0.280
only stroke	111	11	1.22(0.66, 2.25)	0.520	1	0.45(0.06, 3.23)	0.430	10	1.44(0.75, 2.79)	0.280
diabetes and heart diseases	111	6	0.48(0.20, 1.12)	0.090	2	0.99(0.25, 3.88)	0.990	4	0.38(0.13, 1.09)	0.071
diabetes and stroke	14	1	0.68(0.09, 5.27)	0.720	0	–	–	1	0.90(0.11, 7.09)	0.920
heart diseases and stroke	45	3	0.83(0.26, 2.65)	0.750	1	1.44(0.18, 11.4)	0.730	2	0.69(0.17, 2.85)	0.610
diabetes, heart diseases and stroke	11	1	1.30(0.14, 11.9)	0.820	0	–	–	1	1.69(0.16, 17.4)	0.660

^a^ A total of 8,263 people was diagnosed with PCa. Among them, 3,266 were non-invasive cases and 4,997 were invasive cases. Hazard ratios and 95% CIs were calculated using the competitive risk regression model. Model was adjusted for baseline age, education levels, occupation and marital status, body mass index, smoking status, prior PSA test, use of aspirin, AJCC 7^th^ stage, cancer grade and Gleason score.

CMDs, cardiometabolic diseases; CMM, cardiometabolic comorbidities; HR, hazard ratio; CI: confidence interval; PCa, prostate cancer.

### 3.4 Subgroup analysis

We performed the stratified analysis to assess the impact of CMDs on the risks of PCa incidence and mortality across subgroups stratified by age and BMI. Among the elderly population aged ≥ 65 years, baseline presence of a single CMD (HR: 1.11, 95%CI: 1.01~1.20) and CMM (HR: 1.24, 95% CI: 1.02~1.50) were both associated with an elevated risk of incident PCa during the follow-up period. Among participants aged ≥ 65 years, stroke was significantly and positively associated with the incidence of PCa (HR = 1.33, 95% CI: 1.10~1.61). Notably, diabetes was linked to a reduced risk of PCa-specific mortality (HR: 0.52, 95% CI: 0.32~0.85). Regardless of whether participants had a BMI ≥25 kg/m² or not, diabetes was consistently associated with an increased risk of PCa incidence. Furthermore, the combined effect of the CMM triad conferred a more pronounced risk of PCa incidence. In contrast, neither CMD nor CMM showed any significant association with PCa-specific mortality risk (**Supplementary Tables 4**, **S5**).

## 4 Discussion

In this cohort study, we observed that patients with baseline diabetes or stroke is significantly linked to an elevated risk of PCa incidence, with a more pronounced synergistic risk observed in patients with comorbid diabetes and heart disease. Notably, a single CMD confers a protective effect against non-aggressive PCa-specific mortality, whereas no statistically significant association is detected between the three individual conditions (diabetes, heart disease, stroke) and overall PCa-specific mortality.

For the positive association between baseline diabetes, stroke and PCa incidence, as well as the synergistic risk amplification observed in diabetes–cardiac disease comorbidity, these associations may be primarily driven by chronic systemic metabolic disturbances and inflammatory states shared across these cardiometabolic conditions. Diabetes induces persistent hyperglycemia and insulin resistance, which can activate the insulin-like growth factor signaling pathway to promote the proliferation and malignant transformation of prostate epithelial cells (15–18); In addition, high glucose levels can enhance oxidative stress in the prostate microenvironment, further accelerating tumor initiation (19). Stroke, most commonly caused by atherosclerosis, is accompanied by chronic vascular inflammation and endothelial dysfunction, which may create a pro-tumor microenvironment by upregulating pro-inflammatory cytokines (e.g., TNF-α, IL-6) (20) that facilitate angiogenesis and tumor cell survival (21, 22). A recently published study (23) indicated that during the mutual transition and progression between CMDs and cancer, inflammation-related biological pathways exhibit significantly activated characteristics. Specifically, cytokine-cytokine receptor interaction, lysosomal function, Th1/Th2 cell differentiation, and key signaling pathways such as, PI3K-Akt, NF-κB, and IL-17 are particularly active in three stages: “transition from cancer to CMD”, “progression from cancer to death”, and “progression from CMD-cancer multimorbidity to death”. Meanwhile, the study also identified abnormal triglyceride and phospholipid metabolism as key biomarkers for the aforementioned pathological processes. Based on this mechanistic evidence, we hypothesize that when diabetes and heart disease coexist (comorbidity), they synergistically impair the body’s systemic glucose and lipid metabolic balance, disrupting metabolic homeostasis. Furthermore, metabolic disorders further drive a synergistic increase in PCa incidence risk through pathways such as activating the aforementioned inflammatory pathways and perturbing lipid metabolism biomarkers. This combined risk effect may exceed the sum of the risks imposed by diabetes or heart disease acting alone. In contrast, the protective effect of a single CMD on non-aggressive PCa-specific mortality, and the lack of association between the three individual conditions and overall PCa-specific mortality, can be attributed to two interrelated factors. First, this discrepancy is closely linked to the biological heterogeneity of PCa subtypes. Non-aggressive PCa is characterized by low Gleason scores, limited local invasion, and slow progression; patients with a single CMD usually receive standardized chronic disease management and regular clinical follow-up, which not only controls the progression of the primary metabolic disease but also increases the likelihood of early detection of indolent PCa lesions. Timely implementation of active surveillance or minimally invasive interventions for these early-stage tumors can effectively reduce disease-specific mortality. In contrast, overall PCa-specific mortality encompasses deaths from aggressive and metastatic subtypes, which have distinct biological behaviors and are less responsive to the regulatory effects of single CMDs or their associated clinical management strategies. Second, the three individual conditions (diabetes, heart disease, stroke) may exert bidirectional and countervailing effects on PCa progression, which could contribute to the invalid association with overall mortality. For example, while diabetes may promote PCa cell proliferation via hyperglycemia, certain hypoglycemic agents (e.g., metformin) have been reported to exert anti-tumor effects by inhibiting cell cycle progression (24–26); similarly, heart disease-related vascular damage may facilitate tumor angiogenesis, but long-term antiplatelet therapy (e.g., aspirin) could suppress tumor metastasis (27–29). These opposing biological effects neutralize each other when assessing overall PCa-specific mortality, resulting in no statistically significant association.

Public health policies should mandate the inclusion of PCa risk assessment in routine care for patients with CMD comorbidities: For individuals with diabetes-heart disease comorbidity and those with diabetes alone, consideration should be given to lowering the age threshold for PCa screening. For patients with single CMD, prostate-specific antigen testing should be incorporated into annual chronic disease follow-up visits. This stratified integration leverages existing healthcare engagement (a strength of chronic disease management programs) to improve early detection of PCa in high-risk subgroups, while avoiding unnecessary screening burdens for low-risk populations. Importantly, cost-effectiveness analyses are strongly recommended prior to the widespread implementation of these screening measures, such analyses should evaluate factors including the incremental benefit of lowered screening age in diabetes-only vs. diabetes-heart disease populations, and the long-term healthcare resource allocation required for expanded testing. Moreover, data infrastructure and surveillance systems should be enhanced to track CMD-PCa comorbidity trends. For example, surveillance data can assess whether increased screening of diabetes-heart disease comorbidity patients translates to earlier PCa stage at diagnosis, or whether metabolic interventions correlate with reduced PCa incidence over time. Notably, these public health recommendations are derived from observational data and would require prospective validation and evaluation prior to widespread clinical implementation. This data-driven approach ensures that policies are adaptive and evidence-based.

Our study has several limitations. Firstly, the diagnosis of baseline CMDs relied on existing medical records or baseline self-reports, which may have led to misclassification bias, particularly for mild or undiagnosed early-stage metabolic disorders that were not captured in the data. In terms of outcome measurement, the classification of non-aggressive PCa was based on Gleason scores and clinical staging criteria that may have varied slightly across different participating institutions, potentially introducing inter-observer variability that could affect the accuracy of mortality risk analysis. Second, the study cohort was derived from the PLCO Cancer Screening Trial, whose participants were generally more willing to undergo cancer screening and had relatively higher health awareness compared with the general population. This selection bias may limit the extrapolation of our findings to groups with low screening participation rates, as well as to ethnic or demographic subgroups not well-represented in the PLCO cohort. Finally, the study did not fully stratify the analysis by the type, dosage, and duration of medications used for CMD management (e.g., hypoglycemic agents, antiplatelet drugs, lipid-lowering medications). Previous evidence suggests that some of these drugs may exert independent anti-tumor or pro-tumor effects on PCa, which could have interfered with the observed associations between CMDs per se and PCa outcomes. In addition, the study did not assess the impact of changes in CMD status (e.g., diabetes remission, new-onset heart disease) during the follow-up period, which may also modify PCa risk over time.

## 5 Conclusions

Overall, our study underscores the necessity of a personalized, comorbidity-aware approach to PCa prevention and management, offering actionable insights for clinicians, public health policymakers, and researchers alike. Strengths of this study include the large, well-characterized PLCO cohort, long follow-up period (median 17 years), comprehensive assessment of individual and combined CMD exposures, and use of competing risk regression to account for PCa mortality. Future research should employ prospective cohort designs with longer follow-up periods, incorporate genetic and molecular data to explore underlying biological mechanisms, and evaluate the impact of specific CMD treatment regimens on PCa progression. Additionally, studies with more diverse ethnic and demographic populations are needed to validate and extend these findings to broader communities.

## Data Availability

The original contributions presented in the study are included in the article/**Supplementary Material**. Further inquiries can be directed to the corresponding author/s.
