# Supplementary material for: Cardiometabolic comorbidities and prostate cancer risk: evidence from the prostate, lung, colorectal, and ovarian cancer screening trial
Source: Front Oncol. 2026 Apr 29;16:1799790. doi: 10.3389/fonc.2026.1799790 (PMC13167580; doi:10.3389/fonc.2026.1799790)
Supplement: Supplementary file 1 [file DataSheet1.doc]

***Supplemental Materials***

**Table S1.** **Distribution of covariates with missing data before and after imputation in the source population ^a^.**

All the missing values of the variables are less than <5%, so we used the median and the modal value to impute missing values of continuous and categorical variables, respectively. Quantitative variables were imputed by median, and categorical variables were imputed by model value. For model value imputation, we first determined the most frequent category (i.e., pattern) for each variable and then used this pattern to fill in missing values for the corresponding variable. After imputation, the distributions of categorical variables and the means of continuous variables were almost unchanged from their preimputation values.

| **Covariates** | **Before imputation** | **After imputation** | **Number (%) with missing data** |
| --- | --- | --- | --- |
| **Education, n (%)** | | | |
| college below | 27784(38.9) | 27784(38.8) | 155(0.2) |
| college/postgraduate | 43627(61.1) | 43782(61.2) |
| **Occupation, n (%)** | | | |
| working | 31644(44.2) | 31644(44.2) | 286(0.4) |
| retired | 35416(49.5) | 35702(49.9) |
| Others ^b^ | 4220(5.9) | 4220(5.9) |
| **Marital status** | | | |
| married | 59104(82.6) | 59250(82.8) | 143(0.2) |
| widowed | 2585(3.6) | 2585(3.6) |
| divorced | 6483(9.1) | 6483(9.1) |
| separated | 801(1.1) | 801(1.1) |
| never married | 2447(3.4) | 2447(3.4) |
| **Smoking status, n (%)** | | | |
| current | 26163(36.60) | 26163(36.6) | 15(<0.001) |
| former | 8388(11.7) | 8388(11.7) |
| never | 37000(51.7) | 37015(51.7) |
| **Enlarged prostate or BPH** | | | |
| no | 56050(78.3) | 56130(78.4) | 80(0.1) |
| yes | 15436(21.6) | 15436(21.6) |
| **Prior PSA test** | | | |
| no | 32752(45.8) | 32752(45.8) | 10(0.001) |
| yes-one | 25586(35.8) | 25586(35.8) |
| yes-more than one | 6989(9.8) | 6989(9.8) |
| does not know | 6229(8.7) | 6239(8.7) |
| **Family history of cancer, n (%)** | | | |
| no | 34487(48.2) | 34487(48.2) | 177 (0.2) |
| yes | 36902(51.6) | 37079(51.8) |
| **History of hypertension, n (%)** | | | |
| no | 47027(65.7) | 47069(65.8) | 42 (0.1) |
| yes | 24497(34.2) | 24497(34.2) |
| **History of emphysema, n (%)** | | | |
| no | 69378(96.9) | 69422(97.0) | 44(0.1) |
| yes | 2144(3.0) | 2144(3.0) |
| **History of chronic bronchitis, n (%)** | | | |
| no | 68925(96.3) | 68996(96.4) | 71(0.1) |
| yes | 2570(3.6) | 2570(3.6) |
| **AJCC 7^th^ Stage, n (%)** |  |  |  |
| stage I | 2757(33.4) | 2757(3.4) | 7(0.1) |
| stage II | 13(0.2) | 13(0.2) |
| stage IIA | 3294(39.9) | 3301(39.9) |
| stage IIB | 1160(14.0) | 1160(14.0) |
| stage III | 717(8.7) | 717(8.7) |
| stage IV | 315(3.8) | 315(3.8) |
| **Gleason_score** |  |  |  |
| ≤5 | 887(10.7) | 887(10.7) | 118(1.4) |
| 6 | 3598(43.5) | 3716(45.0) |
| 7 | 2672(32.3) | 2672(32.3) |
| ≥8 | 988(12.0) | 988(12.0) |
| **Body mass index, BMI (kg/m^2^)** |  |  |  |
| <18.5 | 247(0.3) | 247(0.3) | 1095(1.5) |
| 18.5 to <25.0 | 18706(26.1) | 18706(26.1) |
| 25.0 to <30.0 | 35499(49.6) | 36594(51.1) |
| ≥30.0 | 16019(22.4) | 16019(22.4) |
| Mean ± SD | 27.50±4.16 | 27.50±4.12 |
| **Pack-years of cigarette smoking, n (%)** |  |  |  |
| Never (0) | 26163(36.6) | 26163(36.6) | 1055 (1.5) |
| >0 and ≤20 | 14080(19.7) | 14080(19.7) |
| >20 | 30268(42.3) | 31323(43.8) |
| Mean ± SD | 25.13±31.47 | 25.32±31.28 |
| ^a^ Values are mean (standard deviation) or counts (percentage) as indicated. | | | |
| ^b^ “Others” refers to homeworker, unemployed, extended sick leave and disabled. | | | |
| Abbreviations: BMI, body mass index; **BPH, benign prostatic hyperplasia.** | | | |

**Table S2 Comparisons of baseline characteristics by prostate cancer cases and noncases in data set ^a^.**

Compared individuals without incident prostate cancer, patients with PCa exhibited the following characteristics: the higher average age, greater educational attainment, the larger proportion of married individuals, the higher percentage of retirees, the greater proportion of individuals who had undergone two or more PSA tests, and a higher prevalence of cancer among first-degree relatives, and a lower prevalence of lung diseases. Furthermore, patients with PCa were more likely to possess a lower BMI and exhibit a higher prevalence of enlarged prostate or BPH.

| **Characteristics** | **Overall** | **No cases** | **PCa** | ***P-value*** |
| --- | --- | --- | --- | --- |
| **N** | **71566** | **63303** | **8263** |  |
| **Baseline ag** | | | | |
| ≤59 | 23060(32.2) | 21112(33.4) | 1948(23.6) | <0.001 |
| 60-64 | 22483(31.4) | 19648(31.0) | 2835(34.3) |  |
| 65-69 | 16591(23.2) | 14280(22.6) | 2311(28.0) |  |
| ≥70 | 9432(13.2) | 8263(13.1) | 1169(14.1) |  |
| (Mean, SD) | (62.68, 5.32) | (62.57, 5.34) | (63.50, 5.07) | <0.001 |
| **Race** | | | | |
| White | 63128(88.2) | 55800(88.1) | 7328(88.7) | 0.254 |
| Non-white | 8395(11.7) | 7463(11.8) | 932(11.3) |  |
| Preferred not to answer | 43(0.1) | 40(0.1) | 3(0.0) |  |
| **Education level** | | | | |
| College below | 27784(38.8) | 24687(39.0) | 3097(37.5) | 0.008 |
| College/postgraduate | 43782(61.2) | 38616(61.0) | 5166(62.5) |  |
| **Occupation** | | | | |
| Working | 31644(44.2) | 28224(44.6) | 3420(41.4) | <0.001 |
| Retired | 35702(49.9) | 31247(49.4) | 4455(53.9) |  |
| Others ^b^ | 4220(5.9) | 3832(6.1) | 388(4.7) |  |
| **Marital status** | | | | |
| Married | 59250(82.8) | 52170(82.4) | 7080(85.7) | <0.001 |
| Widowed | 2585(3.6) | 2298(3.6) | 287(3.5) |  |
| Divorced | 6483(9.1) | 5890(9.3) | 593(7.2) |  |
| Separated | 801(1.1) | 724(1.1) | 77(0.9) |  |
| Never married | 2447(3.4) | 2221(3.5) | 226(2.7) |  |
| **Body mass index, BMI (kg/m^2^)** | | | | |
| <18.5 | 247(0.3) | 227(0.4) | 20(0.2) | <0.001 |
| 18.5 to <25.0 | 18706(26.1) | 16409(25.9) | 2297(27.8) |  |
| 25.0 to <30.0 | 36594(51.1) | 32281(51.0) | 4313(52.2) |  |
| ≥30.0 | 16019(22.4) | 14386(22.7) | 1633(19.8) |  |
| (Mean, SD) | (27.50, 4.12) | (27.53, 4.16) | (27.24, 3.86) | <0.001 |
| **Smoking status** | | | | |
| Current | 26163(36.6) | 22792(36.0) | 3371(40.8) | <0.001 |
| Former | 8388(11.7) | 7636(12.1) | 752(9.1) |  |
| Never | 37015(51.7) | 32875(51.9) | 4140(50.1) |  |
| **Pack-years of cigarette smoking** | | | | |
| Never (0) | 26163(36.6) | 22792(36.0) | 3371(40.8) | <0.001 |
| >0 and ≤20 | 14080(19.7) | 12437(19.6) | 1643(19.9) |  |
| >20 | 31323(43.8) | 28074(44.3) | 3249(39.9) |  |
| **First-degree relatives with cancer** | | | | |
| no | 34487(48.2) | 30735(48.6) | 3752(45.4) | <0.001 |
| yes | 37079(51.8) | 32568(51.4) | 4511(54.6) |  |
| **Aspirin** | | | | |
| no | 34164(47.7) | 30172(47.7) | 3992(48.3) | 0.267 |
| yes | 37402(52.3) | 33131(52.3) | 4271(51.7) |  |
| **Ibuprofen** | | | | |
| no | 54970(76.8) | 48602(76.8) | 6368(77.1) | 0.557 |
| yes | 16596(23.2) | 14701(23.2) | 1895(22.9) |  |
| **Enlarged prostate or BPH** | | | | |
| No | 56130(78.4) | 50000(79.0) | 6130(74.2) | <0.001 |
| yes | 15436(21.6) | 13303(21.0) | 2133(25.8) |  |
| **Prior PSA test** | | | | |
| No | 32752(45.8) | 29225(46.2) | 3527(42.7) | <0.001 |
| Yes-one | 25586(35.8) | 22636(35.8) | 2950(35.7) |  |
| Yes-more than one | 6989(9.8) | 5859(9.3) | 1129(13.7) |  |
| Does not know | 6239(8.7) | 5583(8.8) | 656(7.9) |  |
| **Hypertension** | | | | |
| no | 47069(65.8) | 41554(65.6) | 5515(66.7) | 0.047 |
| yes | 24497(34.2) | 21749(34.4) | 2748(33.3) |  |
| **Lung diseases ^c^** | | | | |
| no | 67397(94.2) | 59516(94.0) | 7881(95.4) | <0.001 |
| yes | 4169(5.8) | 3787(6.0) | 382(4.6) |  |
| **Colon_commodity** | | | | |
| no | 70732(98.8) | 62559(98.8) | 8173(98.9) | 0.493 |
| yes | 834(1.2) | 744(1.2) | 90(1.1) |  |
| **Liver_commodity** | | | | |
| no | 68659(95.9) | 60709(95.9) | 7950(96.2) | 0.180 |
| yes | 2907(4.1) | 2594(4.1) | 313(3.8) |  |
| **Arthritis** | | | | |
| no | 50200(70.1) | 44421(70.23) | 5779(69.9) | 0.662 |
| yes | 21366(29.9) | 18882(29.8) | 2484(30.1) |  |
| **Osteoporosis** | | | | |
| no | 70986(99.2) | 62790(99.2) | 8196(99.23) | 0.997 |
| yes | 580(0.8) | 513(0.8) | 67(0.8) |  |
| ^a^ Values are mean (standard deviation) or counts (percentage) as indicated. | | | | |
| ^b^ “Others” refers to homeworker, unemployed, extended sick leave and disabled. | | | | |
| ^c^ “Lung diseases” refers to asthma, chronic obstructive pulmonary disease (COPD), and bronchiectasis. | | | | |
| **Abbreviations: BMI, body mass index; BPH, benign prostatic hyperplasia, PCa, prostate cancer.** | | | | |

**Table S3 Comparison of pathological characteristics in PCa cases based on baseline CMDs status.**

|  | | **Diabetes** | | | **Heart diseases** | | | **Stroke** | | | **CMD status** | | | |
| --- | --- | --- | --- | --- | --- | --- | --- | --- | --- | --- | --- | --- | --- | --- |
|  |  | **no** | **yes** | ***P-value*** | **no** | **yes** | ***P-value*** | **no** | **yes** | ***P-value*** | **none** | **single CMD** | **CMM** | ***P-value*** |
| **Prostate cancer** | | | | | | | | | | | | | | |
|  | no | 57358  (88.1) | 5845  (90.3) | <0.001 | 54742  (88.2) | 8561  (89.8) | <0.001 | 61547  (88.4) | 1756  (90.7) | <0.001 | 49424  (87.9) | 11669  (89.9) | 2210  (92.4) | <0.001 |
|  | yes | 7733  (11.9) | 530  (9.7) |  | 7291  (11.8) | 972  (10.2) |  | 8082  (11.6) | 182  (9.3) |  | 6772  (12.1) | 1310  (10.1) | 181  (7.6) |  |
| **AJCC 7^th^_Stage** | | | | | | | | | | | | | | |
|  | stage I~II | 2610  (33.8) | 160 (30.2) | 0.008 | 2419 (33.2) | 351 (36.1) | <0.001 | 2702 (33.4) | 68 (37.6) | 0.135 | 2255 (33.3) | 454 (34.7) | 61 (33.7) | <0.001 |
|  | stage IIA | 3104  (40.1) | 197 (37.2) |  | 2945 (40.4) | 356 (36.6) |  | 3236 (40.0) | 65 (35.9) |  | 2752 (40.6) | 485 (37.0) | 64 (35.4) |  |
|  | stage IIB | 1059  (13.7) | 101 (19.1) |  | 997 (13.7) | 163 (16.8) |  | 1127 (13.9) | 33 (18.2) |  | 904 (13.3) | 217 (16.6) | 39 (21.5) |  |
|  | stage III | 668  (8.6) | 49 (9.2) |  | 658 (9.0) | 59 (6.1) |  | 708 (8.8) | 9 (5.0) |  | 609 (9.0) | 99 (7.6) | 9 (5.0) |  |
|  | stage IV | 292  (3.8) | 23 (4.3) |  | 272 (3.7) | 43 (4.4) |  | 309 (3.8) | 6 (3.3) |  | 252 (3.7) | 55 (4.2) | 8 (4.4) |  |
| **Cancer Grade** | | | | | | | | | | | | | | |
|  | grade I | 363  (4.7) | 27 (5.1) | 0.100 | 338 (4.6) | 52 (5.3) | 0.074 | 374 (4.6) | 16 (8.8) | 0.022 | 308 (4.5) | 72 (5.5) | 10 (5.5) | 0.036 |
|  | grade II | 6138  (79.4) | 401 (75.7) |  | 5797 (79.5) | 742 (76.3) |  | 6406 (79.3) | 133 (73.5) |  | 5402 (79.8) | 1003 (76.6) | 134 (74.0) |  |
|  | grade III | 1143  (14.8) | 96 (18.1) |  | 1078 (14.8) | 161 (16.6) |  | 1211 (15.0) | 28 (15.5) |  | 990 (14.6) | 216 (16.5) | 33 (18.2) |  |
|  | grade IV | 41  (0.5) | 5 (0.9) |  | 36 (0.5) | 10 (1.0) |  | 43 (0.5) | 3 (1.7) |  | 31 (0.5) | 12 (0.9) | 3 (1.7) |  |
|  | unknown | 48  (0.6) | 1 (0.2) |  | 42 (0.6) | 7 (0.7) |  | 48 (0.6) | 1 (0.6) |  | 41 (0.6) | 7 (0.5) | 1 (0.6) |  |
| **Gleason score** | | | | | | | | | | | | | | |
|  | ≤5 | 820 (10.6) | 67 (12.6) | <0.001 | 774 (10.6) | 113 (11.6) | 0.013 | 867 (10.7) | 20 (11.0) | 0.822 | 711 (10.5) | 156 (11.9) | 20 (11.0) | 0.002 |
|  | 6 | 3521 (45.5) | 195 (36.8) |  | 3282 (45.0) | 434 (44.7) |  | 3634 (45.0) | 82 (45.3) |  | 3085 (45.6) | 553 (42.2) | 78 (43.1) |  |
|  | 7 | 2494 (32.3) | 178 (33.6) |  | 2389 (32.8) | 283 (29.1) |  | 2618 (32.4) | 54 (29.8) |  | 2210 (32.6) | 411 (31.4) | 51 (28.2) |  |
|  | ≥8 | 898 (11.6) | 90 (17.0) |  | 846 (11.6) | 142 (14.6) |  | 963 (11.9) | 25 (13.8) |  | 766 (11.3) | 190 (14.5) | 32 (17.7) |  |
| **Cancer risk category, n (%)** | | | | | | | | | | | | | | |
|  | Non-aggressive | 3070  (39.7) | 196  (37.0) | 0.232 | 2852  (39.1) | 414  (42.6) | 0.037 | 3182  （39.4） | 84  (46.4) | 0.065 | 2657  (39.2) | 530  (40.5) | 79  (43.6) | 0.368 |
|  | Aggressive | 4663  (60.3) | 334  (63.0) |  | 4439  (60.9) | 558  (57.4) |  | 4900  (60.6) | 97  (53.6) |  | 4115  (60.8) | 780  (59.5) | 102  (56.4) |  |
| **Death status** | | | | | | | | | | | | | | |
|  | no | 4321  (55.9) | 186  (35.1) | <0.001 | 4110  (56.4) | 397  (40.8) | <0.001 | 4455  (55.1) | 52  (28.7) | <0.001 | 3923  (57.9) | 535  (40.8) | 49  (27.1) | <0.001 |
|  | yes | 3412  (44.1) | 344  (45.5) |  | 3181  (43.6) | 575  (59.2) |  | 3627  (44.9) | 129  (71.3) |  | 2849  (42.1) | 775  (59.2) | 132  (72.9) |  |
| Values are mean ± standard deviation for continuous variables and counts (percentage) for categorical variable as indicated. | | | | | | | | | | | | | | |
| Abbreviations: CMD, **cardiometabolic diseases; CMM, cardiometabolic comorbidities; PCa, prostate cancer** | | | | | | | | | | | | | | |

**Table S4 Age subgroup analyses on the associations of CMDs and PCa- incidence and specific mortality.**

| **Exposure** | | **PCa-incidence** | | | | **PCa-specific mortality** | | | |
| --- | --- | --- | --- | --- | --- | --- | --- | --- | --- |
|  |  | **Age < 65** | | **Age ≥ 65** | | **Age < 65** | | **Age ≥ 65** | |
|  |  | ***HR (95% CI)*** | ***P-value*** | ***HR (95% CI)*** | ***P-value*** | ***HR (95% CI)*** | ***P-value*** | ***HR (95% CI)*** | ***P-value*** |
| **Diabetes** | | 1.13(0.99, 1.28) | 0.074 | 1.23(1.09, 1.39) | 0.001 | 1.10(0.67, 1.82) | 0.710 | 0.52(0.32, 0.85) | 0.009 |
| **Heart diseases** | | 0.92(0.83, 1.02) | 0.102 | 1.00(0.91, 1.10) | 0.954 | 1.18(0.77, 1.81) | 0.440 | 0.91(0.66, 1.26) | 0.570 |
| **Stroke** | | 0.88(0.69, 1.12) | 0.296 | 1.33(1.10, 1.61) | 0.003 | 0.93(0.36, 2.40) | 0.890 | 1.15(0.62, 2.14) | 0.660 |
| **CMD status** | |  | | | | | | | |
|  | none | 1.0(reference) |  | 1.0(reference) |  | 1.0(reference) |  | 1.0(reference) |  |
|  | single CMD | 0.96(0.88, 1.05) | 0.334 | 1.11(1.02, 1.20) | 0.018 | 1.23(0.87, 1.74) | 0.250 | 0.88(0.67, 1.17) | 0.390 |
|  | CMM | 1.02(0.81, 1.29) | 0.848 | 1.24(1.02, 1.50) | 0.033 | 0.92(0.35, 2.39) | 0.870 | 0.46(0.19, 1.07) | 0.071 |
| **CMD combination** | |  | | | | | | | |
|  | none | 1.0(reference) |  | 1.0(reference) |  | 1.0(reference) |  | 1.0(reference) |  |
|  | only diabetes | 1.12(0.96, 1.29) | 0.139 | 1.21(1.05, 1.40) | 0.009 | 1.19(0.69, 2.07) | 0.530 | 0.58(0.34, 1.00) | 0.051 |
|  | only heart diseases | 0.89(0.80, 0.99) | 0.039 | 0.99(0.90, 1.11) | 0.986 | 1.29(0.82, 2.03) | 0.270 | 0.98(0.70, 1.39) | 0.930 |
|  | only stroke | 0.93(0.68, 1.26) | 0.640 | 1.64(1.29, 2.09) | <0.001 | 0.92(0.25, 3.36) | 0.890 | 1.30(0.64, 2.66) | 0.470 |
|  | diabetes and heart diseases | 1.24(0.92, 1.67) | 0.155 | 1.38(1.08, 1.76) | 0.011 | 0.86(0.24, 3.10) | 0.820 | 0.33(0.13, 1.08) | 0.067 |
|  | diabetes and stroke | 0.56(0.26, 1.18) | 0.128 | 0.85(0.41,1.80) | 0.677 | 1.54(0.22, 10.70) | 0.660 | - | - |
|  | heart diseases and stroke | 0.85(0.53, 1.38) | 0.519 | 1.02(0.70, 1.48) | 0.926 | 0.85(0.12, 5.95) | 0.870 | 0.75(0.17, 3.32) | 0.700 |
|  | diabetes, heart diseases and stroke | 1.63(0.61, 4.37) | 0.328 | 1.48(0.70, 3.12) | 0.302 | - | - | 1.57(0.15, 16.00) | 0.700 |
| N=8263. Hazard ratios and 95% CIs were calculated using the Cox proportional hazards regression model and competitive risk regression model. Model with prostate cancer incidence was adjusted for baseline age, education levels, occupation and marital status, body mass index, smoking status, first-degree relatives with cancer, prior PSA test and history of enlarged prostate or BPH, hypertension and lung diseases. Model with prostate cancer survivor’ death was adjusted for baseline age, education levels, occupation and marital status, body mass index, smoking status, prior PSA test, use of aspirin, AJCC 7^th^ stage, cancer grade and Gleason score. | | | | | | | | | |
| Abbreviations: CMD, **cardiometabolic diseases; CMM, cardiometabolic comorbidities; HR, hazard ratio; CI: confidence interval.** | | | | | | | | | |

**Table S5** **BMI subgroup analyses on the associations of CMDs and PCa-incidence and specific mortality.**

| **Exposure** | | **PCa-incidence** | | | | **PCa-specific mortality** | | | |
| --- | --- | --- | --- | --- | --- | --- | --- | --- | --- |
|  |  | **BMI < 25 kg/m^2^** | | **BMI ≥ 25 kg/m^2^** | | **BMI < 25 kg/m^2^** | | **BMI ≥ 25 kg/m^2^** | |
|  |  | ***HR (95% CI)*** | ***P*** | ***HR (95% CI)*** | ***P*** | ***HR (95% CI)*** | ***P*** | ***HR (95% CI)*** | ***P*** |
| **Diabetes** | | 1.36(1.07, 1.72) | 0.013 | 1.16(1.05, 1.28) | 0.002 | 0.83(0.35, 1.96) | 0.670 | 0.69(0.47, 1.01) | 0.056 |
| **Heart diseases** | | 1.03(0.94, 1.13) | 0.962 | 0.96(0.89, 1.04) | 0.301 | 0.78(0.43, 1.43) | 0.430 | 1.03(0.77, 1.37) | 0.850 |
| **Stroke** | | 1.54(1.15, 2.06) | 0.004 | 1.03(0.87, 1.23) | 0.701 | 0.27(0.04, 2.03) | 0.210 | 1.35(0.80, 2.29) | 0.270 |
| **CMD status** | |  | | | | | | | |
|  | none | 1.0(reference) |  | 1.0(reference) |  | 1.0(reference) |  | 1.0(reference) |  |
|  | single CMD | 1.08(0.96, 1.23) | 0.199 | 1.01(0.95, 1.09) | 0.707 | 0.88(0.53, 1.45) | 0.620 | 1.01(0.79, 1.30) | 0.930 |
|  | CMM | 1.34(0.90, 1.99) | 0.145 | 1.10(0.93, 1.29) | 0.272 | - | - | 0.69(0.36, 1.33) | 0.270 |
| **CMD combination** | |  | | | | | | | |
|  | none | 1.0(reference) |  | 1.0(reference) |  | 1.0(reference) |  | 1.0(reference) |  |
|  | only diabetes | 1.33(1.02, 1.74) | 0.035 | 1.15(1.03, 1.29) | 0.013 | 1.06(0.44, 2.53) | 0.900 | 0.74(0.48, 1.15) | 0.180 |
|  | only heart diseases | 1.01(0.87, 1.16) | 0.900 | 0.94(0.86, 1.02) | 0.125 | 0.90(0.49, 1.66) | 0.750 | 1.11(0.81, 1.52) | 0.510 |
|  | only stroke | 1.62(1.14, 2.30) | 0.007 | 1.19(0.96, 1.50) | 0.114 | 0.37(0.05, 2.80) | 0.330 | 1.55(0.82, 2.93) | 0.180 |
|  | diabetes and heart diseases | 1.45(0.82, 2.58) | 0.200 | 1.28(1.05, 1.56) | 0.016 | - | - | 0.55(0.23, 1.32) | 0.180 |
|  | diabetes and stroke | 2.16(0.54, 8.68) | 0.276 | 0.60(0.34, 1.07) | 0.082 | - | - | 0.71(0.09, 5.50) | 0.750 |
|  | heart diseases and stroke | 1.26(0.69, 2.27) | 0.454 | 0.93(0.66, 1.30) | 0.662 | - | - | 1.08(0.34, 3.50) | 0.890 |
|  | diabetes, heart diseases and stroke | 9.63(1.35, 68.70) | 0.024 | 1.37(0.74, 2.56) | 0.318 | - | - | 1.42(0.15, 13.20) | 0.760 |
| N=8263. Hazard ratios and 95% CIs were calculated using the Cox proportional hazards regression model and competitive risk regression model. Model with prostate cancer incidence was adjusted for baseline age, education levels, occupation and marital status, body mass index, smoking status, first-degree relatives with cancer, prior PSA test and history of enlarged prostate or BPH, hypertension and lung diseases. Model with prostate cancer survivor’ death was adjusted for baseline age, education levels, occupation and marital status, body mass index, smoking status, prior PSA test, use of aspirin, AJCC 7^th^ stage, cancer grade and Gleason score. | | | | | | | | | |
| Abbreviations: CMD, **cardiometabolic diseases; CMM, cardiometabolic comorbidities; HR, hazard ratio; CI: confidence interval.** | | | | | | | | | |
